# A Cadaveric Case Report of a Quadricuspid Pulmonary Valve and Its Clinical Implications

**DOI:** 10.7759/cureus.111566

**Published:** 2026-06-26

**Authors:** Sara Omari, Buvaneshwari Sathishkumar, Huiying Yang, Ahmad Imam, Kamal A Abouzaid

**Affiliations:** 1 Department of Anatomical Sciences, William Carey University College of Osteopathic Medicine, Hattiesburg, USA

**Keywords:** congenital anomaly, heart defect, pulmonary valve, qpv, quadricuspid pulmonary valve

## Abstract

A quadricuspid pulmonary valve (QPV) is a rare congenital anomaly resulting from abnormal semilunar valvulogenesis during embryonic development. Most reported cases are identified incidentally at autopsy, and the condition remains likely underrecognized clinically because of the pulmonary valve’s retrosternal location and limited visualization on transthoracic echocardiography. We report a cadaveric case of a Hurwitz type F QPV identified during a routine dissection of a 78-year-old female donor. Gross examination revealed two larger cusps and two unequal smaller anterior cusps associated with mild dilation of the pulmonary trunk. No evidence of pulmonary stenosis, right ventricular hypertrophy, or pulmonary artery aneurysm (PAA) was observed. This report reviews the embryologic basis, morphologic classification, diagnostic challenges, clinical associations, and management considerations of QPV. Although many QPVs remain asymptomatic, the anomaly has been associated with congenital cardiac defects, pulmonary regurgitation, PAA, and rare surgical implications, including use in the Ross procedure. Recognition of QPV variants is important for accurate diagnosis, risk assessment, and individualized surgical planning. We aim to contribute to the currently limited body of literature relating to this uncommon anomaly with cadaveric insights of one of the rarest variations of the QPV.

## Introduction

The pulmonary valve, located between the right ventricle and pulmonary artery, carries deoxygenated blood away from the heart. It is a crescent-shaped, tricuspid leaflet that permits blood flow into the pulmonary artery during systolic contraction of the heart while preventing backflow into the right ventricle during diastole [1]. The embryological development of the pulmonary valve begins by the fourth week of gestation, when a pair of mesenchymal truncoconal ridges form within the truncus arteriosus [2,3]. These ridges undergo spiral growth and fusion to form the aorticopulmonary septum, separating the outflow tract into the aorta and pulmonary trunk [2-4]. Subsequently, semilunar valves arise from the proliferation and remodeling of the endocardial cushion-derived mesenchymal tissue at the junction of the truncus arteriosus and conus cordis, giving rise to the crescent-shaped valve cusps [2,4]. According to Hurwitz and Roberts, the formation of an aberrant semilunar valve could be due to abnormal fusion of the aorticopulmonary septum or abnormal mesenchymal proliferations in the common trunk [2].

A quadricuspid pulmonary valve (QPV) is a rare congenital anomaly where the pulmonary valve contains four cusps instead of the typical three, resulting from abnormal valvulogenesis during embryologic development. It was first described by Hurwitz and Roberts, who classified seven subtypes (A-G) based on the relative size and shape of the cusps [2]. Among 121 reported cases of QPV, the two most common variations were type B (three equal cusps and one smaller cusp) and type C (two equal larger and two equal smaller), accounting for approximately 60% (72 valves) and 15% (18 valves) of occurrence rates, respectively [2]. In a subset of 79 cases with reported sex distribution, Hurwitz and Roberts observed a male-to-female ratio of 2:1 (52 males and 27 females) [2]. Furthermore, the estimated incidence of QPV ranges from 1 in 400 to 1 in 2,000 based on the findings of Ingham and Simonds respectively [5,6]. More recently, a European donor heart study reported a QPV prevalence of 0.2% (8 of 3,861 hearts [7]. These findings indicate that relatively few cases of QPV have been formally documented, emphasizing that it is both rare and likely underreported. We present a cadaveric case of QPV type F variant, characterized by two large cusps and two unequal smaller cusps. Our aim is to contribute to the limited literature on this rare morphological variant of QPV by comparing our findings with previously reported cases and reviewing its potential clinical implications. 

## Case presentation

During the summer prosection seminar at William Carey University College of Osteopathic Medicine, a 78-year-old female cadaveric donor obtained through the University of South Alabama Anatomical Gift Program was dissected. The cause of death was documented as a malignant neoplasm of the greater curvature of the stomach.

**Figure 1:**
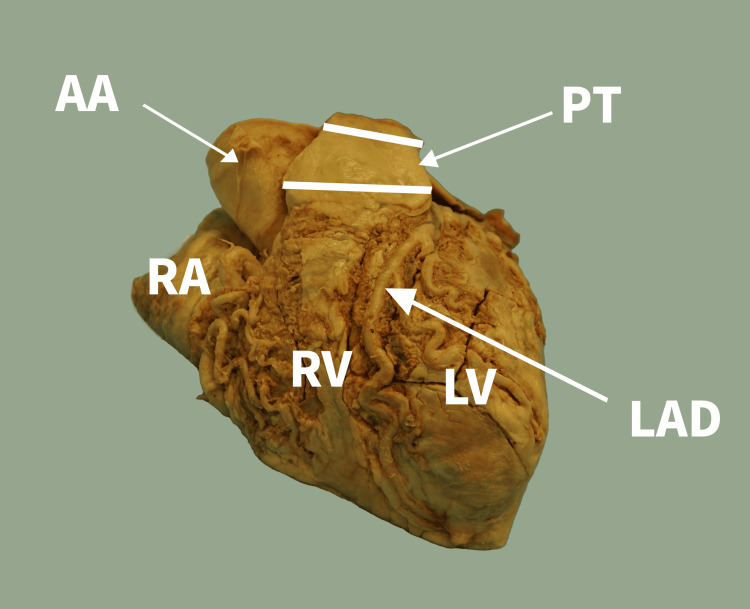
Anterior view of external features of the cadaveric heart demonstrating mild dilation of the pulmonary trunk root AA: aortic arch; PT: pulmonary trunk; RA: right atrium; RV: right ventricle; LV: left ventricle; LAD: left anterior descending artery

During dissection of the external and internal features of the heart, a slight dilation of the root of the pulmonary trunk was noted (Figure 1).

**Figure 2:**
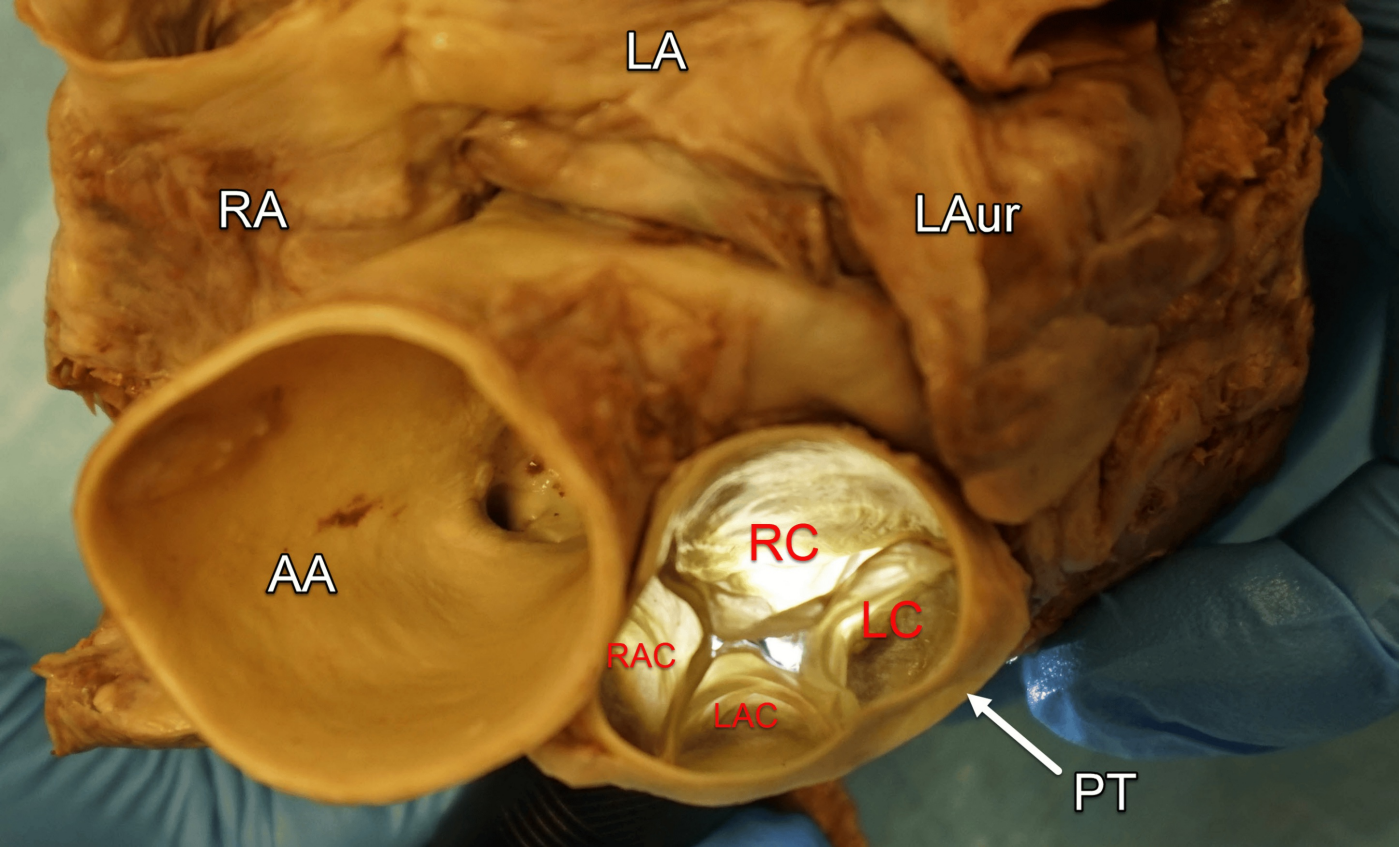
Quadricuspid pulmonary valve demonstrating division of the anterior cusp into right anterior (RAC) and left anterior (LAC) cusps from the superior (bird's eye) perspective AA: aortic arch; PT: pulmonary trunk; RA: right atrium; LA: left atrium; LAur: left auricle; RC: right cusp; LC: left cusp; RAC: right anterior cusp; LAC: left anterior cusp

**Figure 3:**
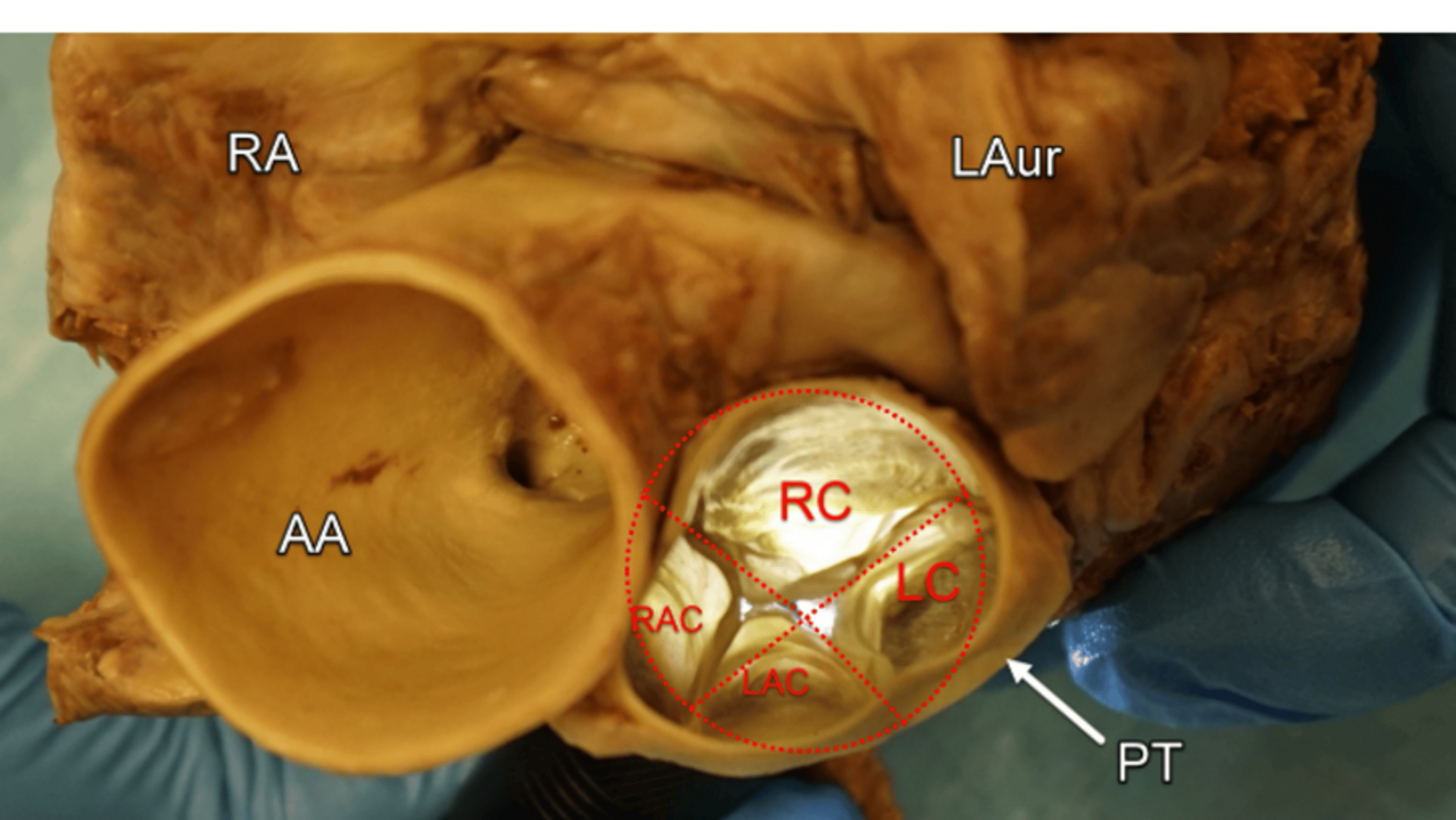
Schematic representation superimposed on the pulmonary trunk showing the QPV from a superior (bird’s-eye) perspective AA: aortic arch; PT: pulmonary trunk; RA: right atrium; LAur: left auricle; RC: right cusp; LC: left cusp; RAC: right anterior cusp; LAC: left anterior cusp

The pulmonary trunk was then transected according to standard anatomical dissection techniques. Initial inspection of the pulmonary valve from a superior view revealed four semilunar cusps compressed against the vessel wall by a firm blood clot. Following removal of the clot, further examination demonstrated that the valve included the typical right and left cusps; however, the usual single anterior cusp was replaced by two smaller anterior cusps. For descriptive clarity, these were designated as the right anterior cusp (RAC) and left anterior cusp (LAC) (Figure 2). A schematic representation was superimposed on the pulmonary trunk to illustrate these findings from a superior (bird’s-eye) perspective (Figure 3).

**Figure 4:**
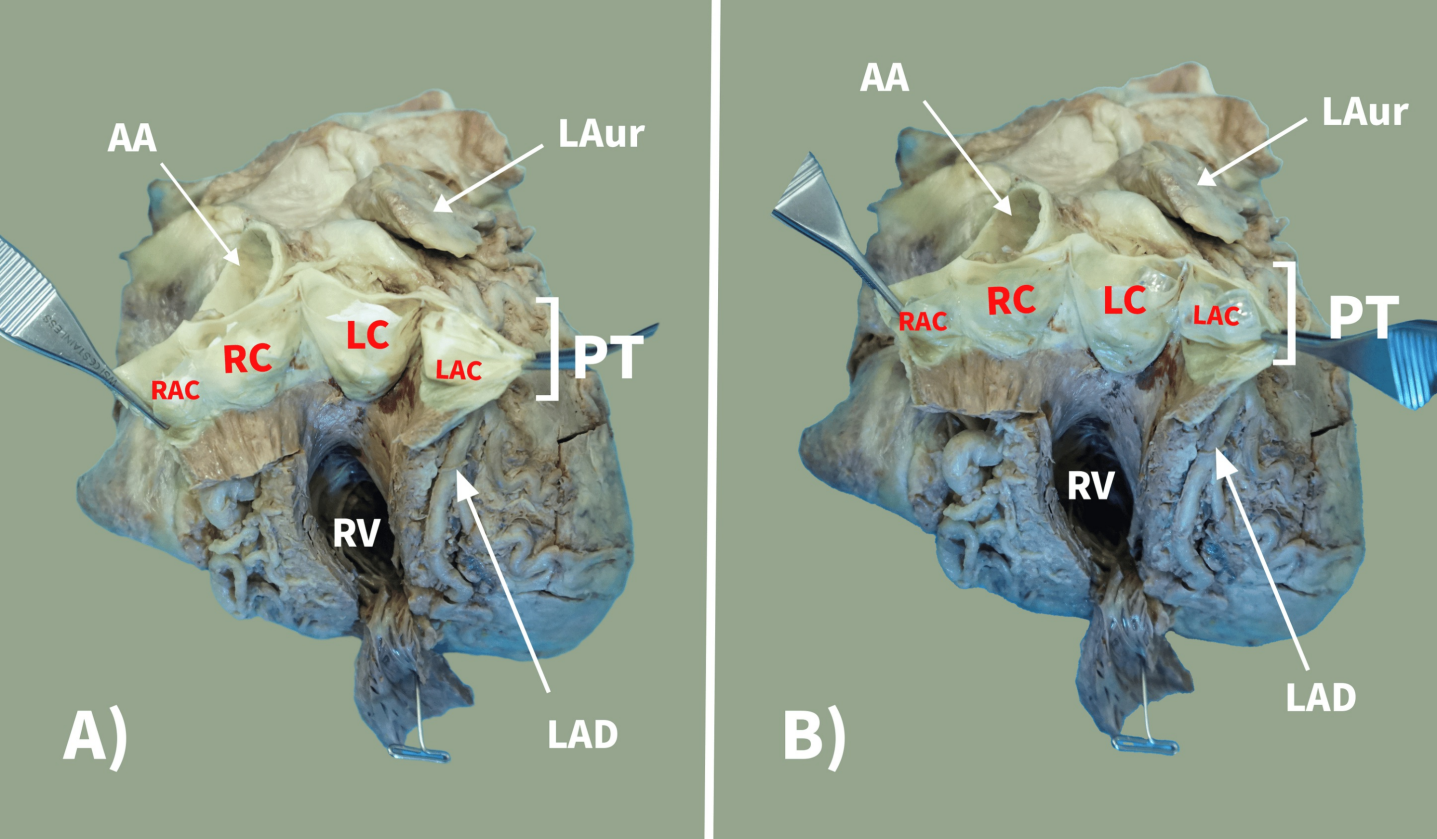
(A) Incised pulmonary trunk demonstrating all cusps following a vertical incision between right anterior cusp (RAC) and left anterior cusp (LAC), with cotton balls inserted to enhance visualization. (B) Incised pulmonary trunk with small beads inserted into each cusp AA: aortic arch; PT: pulmonary trunk; LAur: left auricle; RC: right cusp; LC: left cusp; RAC: right anterior cusp; LAC: left anterior cusp; RV: right ventricle; LAD: left anterior descending artery

To better evaluate cusp morphology in situ, the pulmonary trunk was incised circumferentially at the level of the lunules. A vertical incision was then made between the RAC and LAC, allowing all cusps to be displayed side by side. Cotton balls (Figure 4A) and small beads (Figure 4B) were inserted into each cusp to enhance visualization. Notably, the LAC accommodated approximately two beads, whereas the RAC accommodated only one bead, indicating asymmetry in cusp size. This difference corresponded to a relatively larger sinus of Valsalva associated with the LAC compared to the RAC.

Aside from the QPV, the remaining cardiac valves appeared grossly normal, with only mild calcification noted in the atrioventricular valves. There was no evidence of pulmonary valve stenosis or right ventricular hypertrophy. Although a slight dilation of the root of the pulmonary trunk was observed, interpretation is limited due to the presence of a postmortem clot and potential formalin-induced tissue distortion.

This case illustrates a rare variant of a QPV, most likely arising from abnormal embryologic cusp formation, and contributes to the limited body of literature describing the morphology and developmental basis of this uncommon anomaly.

## Discussion

In this case study, we examine a rare presentation of a QPV with two large valves and two unequal smaller valves (Hurwitz type F). While QPV is most commonly an incidental finding on autopsy, several methods of clinical diagnosis have been identified [8]. The transthoracic echocardiogram (TTE), cardiac magnetic resonance (CMR), and contrast computed tomography (CT) are the diagnostic methods used [9]. Anatomically, QPV is in a retrosternal location, making it hard to access with TTE [10]. Previous reports indicate that type A (four equal cusps) QPVs are more commonly diagnosed with TTE than type B [9]. By contrast, types B (three equal large cusps and one small cusp) and C (two equal large, two equal small) are usually diagnosed at autopsy [9]. In one case, QPV diagnosis was made more obvious due to concurrent pulmonary dilatation [9]. Our case was a type F QPV, which is relatively similar in shape to type C except that the two smaller valves in type F are different sizes. We suspect that type F variation likewise carries a similar diagnostic challenge.

Supernumerary cusps are often clinically silent, but are associated with other conotruncal abnormalities, such as aortic atresia with interrupted aortic arch, patent ductus arteriosus, ventricular septal defects, aortic valve issues, atrial septal defects, pulmonary valvular stenosis, valvular regurgitation, and pulmonary artery aneurysm (PAA) [9,11-13]. Aortic valve defects are often associated with pulmonary valve defects [14]. Both stenosis and congenital heart abnormalities are more common in QPVs than quadricuspid aortic valves (QAVs), but QPVs are less likely to be associated with infective endocarditis [9]. Hurwitz et al. reported that 6 (4%) of 158 QPV cases reviewed functioned abnormally, and 2 of these valves were stenotic [2].

Furthermore, pulmonary regurgitation is another common feature of QPV. In regurgitant QAVs, differences in cusp size may increase the likelihood of regurgitation due to hemodynamic instability [15]. We speculate that this may also be the case for QPVs with unequal cusp sizes, as in the present case. Additionally, two-thirds of QPVs have hypoplastic cusps, which could lead to pulmonary regurgitation. Subsequent pulmonary annular dilatation may worsen and progressively increase the pulmonary regurgitation, which could lead to PAA [13,14]. QPV regurgitation can also cause increased right ventricular preload, which may lead to right ventricular dysfunction and hypertrophy with pulmonary artery dilation [16]. Our case had a mildly dilated pulmonary trunk as shown in Figure 1, but there was no evidence of right ventricular hypertrophy or PAA, so the QPV has no morphologic evidence of hemodynamic consequence (no stenosis, right ventricular hypertrophy, or aneurysm).

The QPV has been implicated in several surgical procedures. The use of the QPV in the Ross procedure was first reported by Sommer et al., where a QPV was used as an autograft replacement of the aortic valve [17]. The use of a QPV for the Ross procedure is controversial, so it is important to consider the anatomy of the QPV as well as patient factors (such as age, comorbidities, etc.) and also to take care to maintain commissural symmetry to avoid cusp prolapse [18].

Symptomatic QPVs have been treated with tricuspidization, bicuspidization, prosthetic valve replacement, percutaneous balloon valvuloplasty, and transcatheter pulmonary valvuloplasty with an Inoue balloon catheter [13,16,19-21]. In the case of a symptomatic regurgitant QPV, the valve can be repaired with either a prosthetic valve replacement or native valve repair. Because prosthetic valve replacement requires anticoagulant therapy, native valve repair is often chosen over prosthetic placement [16].

For QPVs with PAA, additional surgeries should be considered. An arterioplasty was selected to repair an aneurysm due to the determination that the pulmonary regurgitation secondary to the QPV was the major contributor to the PAA, and the probability of arterial structural abnormalities was deemed to be low [13]. It is important to consider surgery in QPV cases with other PAA risk factors [22,23].

## Conclusions

This case describes a rare Hurwitz type F QPV identified during cadaveric dissection. The finding contributes to the limited literature on QPV morphology and highlights the diagnostic challenges associated with this uncommon anomaly. Although often clinically silent, QPVs may be associated with valvular dysfunction, congenital cardiac abnormalities, pulmonary artery dilation, and aneurysm formation. Awareness of anatomic variation in QPV is important for clinicians, radiologists, and surgeons, particularly when considering imaging interpretation or surgical intervention. Continued documentation of QPV variants may improve understanding of their embryologic development, clinical significance, and long-term outcomes.
